# Circulation of Lassa virus across the endemic Edo-Ondo axis, Nigeria, with cross-species transmission between multimammate mice

**DOI:** 10.1080/22221751.2023.2219350

**Published:** 2023-06-08

**Authors:** Adetunji Samuel Adesina, Akinlabi Oyeyiola, Adeoba Obadare, Joseph Igbokwe, Chukwuyem Abejegah, Patience Akhilomen, Umaru Bangura, Danny Asogun, Ekaete Tobin, Olufemi Ayodeji, Omolaja Osoniyi, Chris Davis, Emma C. Thomson, Meike Pahlmann, Stephan Günther, Elisabeth Fichet-Calvet, Ayodeji Olayemi

**Affiliations:** a Department of Biochemistry and Molecular Biology, Obafemi Awolowo University, Ile Ife, Osun State, Nigeria; b Natural History Museum, Obafemi Awolowo University, Ile Ife, Osun State, Nigeria; c Department of Zoology, Obafemi Awolowo University, Ile Ife, Osun State, Nigeria; d Federal Medical Centre, Owo, Ondo State, Nigeria; e Irrua Specialist Teaching Hospital, Irrua, Edo State, Nigeria; f Bernhard Nocht Institute for Tropical Medicine, Hamburg, Germany; g Centre for Virus Research, University of Glasgow, Glasgow, UK

**Keywords:** Lassa virus, *Mastomys*, host-switching, emergence and spreading, Nigeria

## Abstract

We phylogenetically compared sequences of the zoonotic Lassa virus (LASV) obtained from *Mastomys* rodents in seven localities across the highly endemic Edo and Ondo States within Nigeria. Sequencing 1641 nt from the S segment of the virus genome, we resolved clades within lineage II that were either limited to Ebudin and Okhuesan in Edo state (2g-beta) or along Owo-Okeluse-Ifon in Ondo state (2g-gamma). We also found clades within Ekpoma, a relatively large cosmopolitan town in Edo state, that extended into other localities within Edo (2g-alpha) and Ondo (2g-delta). LASV variants from *M. natalensis* within Ebudin and Ekpoma in Edo State (dated approximately 1961) were more ancient compared to those from Ondo state (approximately 1977), suggesting a broadly east-west virus migration across south-western Nigeria; a pattern not always consistent with LASV sequences derived from humans in the same localities. Additionally, in Ebudin and Ekpoma, LASV sequences between *M. natalensis* and *M. erythroleucus* were interspersed on the phylogenetic tree, but those from *M. erythroleucus* were estimated to emerge more recently (approximately 2005). Overall, our results show that LASV amplification in certain localities (reaching a prevalence as high as 76% in Okeluse), anthropogenically-aided spread of rodent-borne variants amidst the larger towns (involving communal accommodation such as student hostels), and virus-exchange between syntopic *M. natalensis* and *M. erythroleucus* rodents (as the latter, a savanna species, encroaches southward into the degraded forest) pose perpetual zoonotic hazard across the Edo-Ondo Lassa fever belt, threatening to accelerate the dissemination of the virus into non endemic areas.

## 1. Introduction

**Figure 1.**
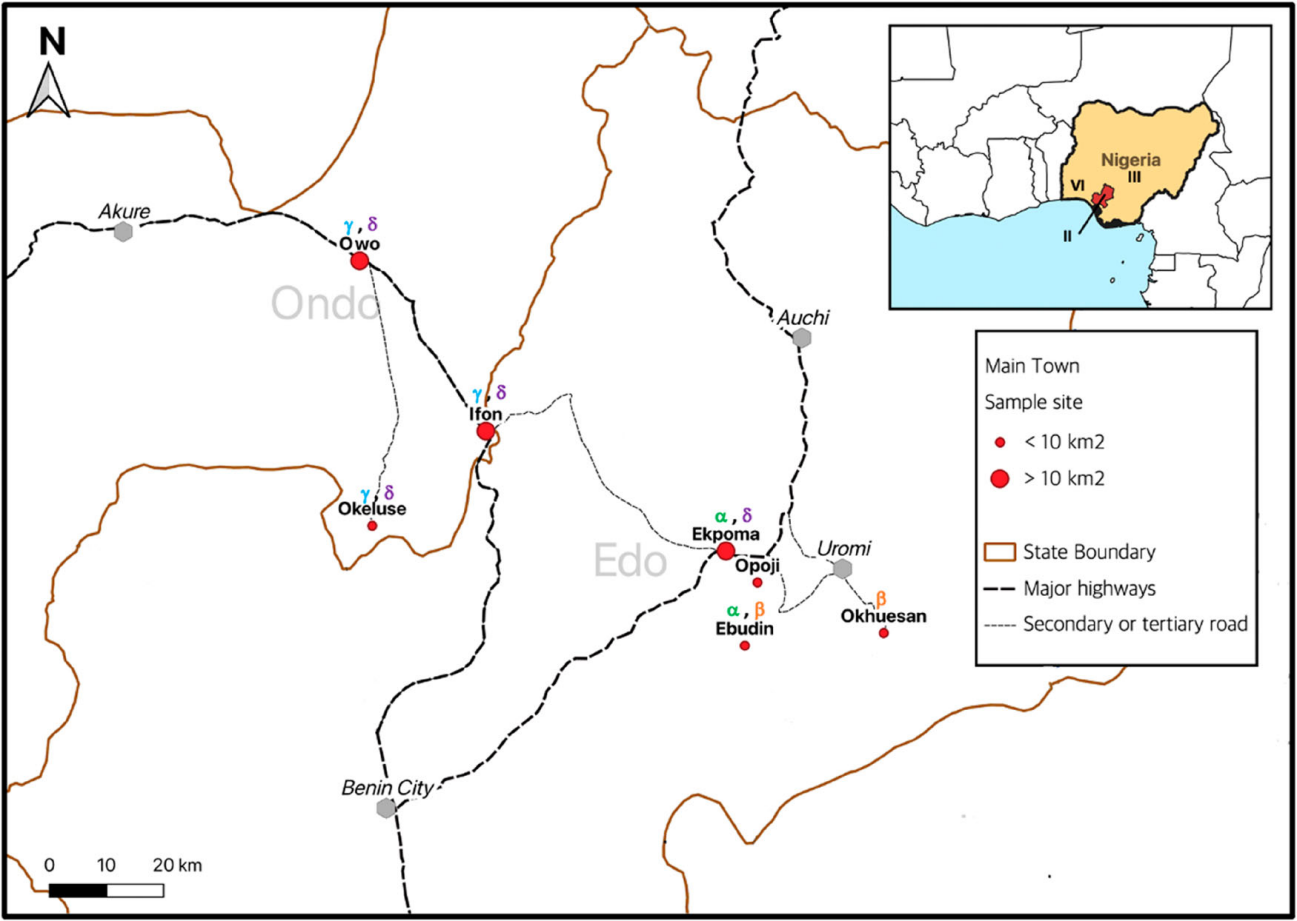
The Edo-Ondo Lassa fever belt. Red dots within the enlarged map indicate localities sampled in this study. Above each locality are stated the LASV clades resolved within sub-lineage 2 g (i.e. 2gα, 2gβ, 2gγ & 2gδ). Inserted is Nigeria with the region comprising Edo and Ondo States shaded red. Roman numerals within Nigeria denote geographic distribution of LASV lineages that, leading up to this study, were detected within Nigeria in the rodents *Mastomys natalensis* (lineage II), *Mastomys erythroleucus* (III) and *Hylomyscus pamfi* (VI) respectively.

Annually across Western Africa, Lassa fever, a lethal haemorrhagic ailment, is estimated to infect 897,700 people and projected to kill 18,000 [1]. Nigeria, where the case-fatality ratio reaches up to 20%, accounts for more than half of the diagnoses [2]. Within southern Nigeria, the Lassa fever belt that comprises northern Edo State, Ose Local Government Area in Ondo State on the border with Edo state, and localities further west within Ondo State such as Owo (Figure 1), is considered the most endemic, accounting for up to 71% of total cases within the country [3–5]. The zoonotic Lassa virus (LASV), which causes Lassa fever in humans, is maintained in nature by rodent reservoirs [6]. Humans contract the virus when exposed to the excreta of viruric rodents [7]. LASV is currently ranked amongst viruses with the highest risk of animal-to-human spillover and is also among the likeliest to emerge outside its endemic zone [8].

Describing the circulation of LASV genetic diversity across the Edo-Ondo area is vital to improved epidemiology of the disease and will similarly be helpful to interventions that include targeted rodent control [9] and vaccine development [10]. Moreover, because of the high variability of the LASV genome, diagnostics have to be periodically updated as novel sequences continue to be encountered [11]. Presently, various LASV lineages are distributed across the Western African region: I, the prototypic LASV strain, detected in Lassa village, north-eastern Nigeria, but apparently extinct; II in southern Nigeria; III in central Nigeria; IV in Guinea and Sierra Leone; V in northern Cote d’Ivoire and southern Mali; VI in south-western Nigeria; and VII in Benin and Togo [12–14].

Hitherto, genetic sequences representing LASV variety in the Edo-Ondo axis have typically been generated from humans; particularly during the course of Lassa fever diagnosis at the Irrua Specialist Teaching Hospital (ISTH) in Edo State [15–17]. At ISTH, examining a short LASV glycoprotein fragment (≈ 300 bp.), Asogun et al. [17] detected lineage II in PCR-positive human patients from Edo and Ondo States, in agreement with previous surveys. Within this lineage, they resolved 3 phylogenetic clades that corresponded only to a limited extent to the locality of origin of the LASV variants. Other investigations [15,16,18,19] likewise found lineage II variants circulating across various localities within southern Nigeria. Their phylogenetic analyses largely indicated short chains of human infection, each apparently initiated by contact with a rodent. Also, using a dataset that contained mostly human-derived sequences in a current phylogeography of LASV across Nigeria, Ehichioya et al. [20] resolved various sub-lineages within lineage II, with the clade “2g” peculiar to Edo and Ondo states. Their analyses also indicated westward movement of the virus frontier through Ondo State. These authors on recent LASV epidemiology and phylogeography recommend intensified ecological sampling to increase the complement of rodent-derived LASV sequences available from Nigeria in genetic databases.

LASV variants obtained from rodents can be regarded as more reliable for phylogeographic studies than those obtained from humans. Rodents from the genus *Mastomys*, on their own, do not normally wander beyond small home ranges found to be generally less than 100 m in radius [21,22]. Therefore, LASV-positive rodents are likely to have obtained their infection from the site they were captured. On the other hand, people might travel between towns and villages or provide imprecise information, resulting in disparities between their assumed and actual sites of exposure [23].

Across Western Africa, the Natal multimammate mouse (“*Mastomys natalensis*” Smith, 1834) is the main LASV reservoir [6,24]. Correspondingly, Olayemi et al. [25] detected LASV lineage II in *M. natalensis* within Ekpoma and Eguare Egoro in Edo State. Already, comparing these LASV sequences to those obtained from humans also in Ekpoma and its environs demonstrate that those from humans are generally more ancient; calling for a broader, bi-directional view of LASV epidemiology that includes reverse zoonosis [26]. Additionally, Olayemi et al. [27] discovered new LASV rodent reservoirs outside the Edo-Ondo zone: with lineage III of the virus in the Guinea multimammate mouse (“*M. erythroleucus*” Temminck, 1853) in Onmba Abena, Central Nigeria, and a novel lineage (VI) in the “African wood mouse” (*Hylomyscus pamfi* [27]) within Kako, south-western Nigeria (Figure 1). This created the impression that different rodent hosts harbour separate LASV lineages around Nigeria. A follow-up serological study by Olayemi et al. [28] found 1 *M. erythroleucus* IgG-positive for LASV in Ekpoma, the same locality lineage II was detected in *M. natalensis*. This raised the questions: is *M. erythroleucus* also an active LASV host within Ekpoma or other parts of Edo State? If so, what lineage of the virus does it carry in this area?

The initial reports we have made on LASV ecology in Edo state [25,28,29] are based on rodent trapping carried out during 2011–2012. Here, with expanded sampling through 2014–2019, our objective is to describe the genetic and geographic variation between LASV variants obtained from *Mastomys* populations across the Edo-Ondo Lassa fever belt.

## 2. Materials and methods

### 2.1. Study area and trapping

Our study area consists of seven localities forming a longitudinal gradient east-to-west from northern Edo State (Ebudin N 6° 35’ 48.4 E 6°10’ 53.3”, Ekpoma N 6° 44’ 29.1” E 6° 06’ 17.6”, Okhuesan N 6°36’ 40.2” E 6° 24’ 15.2”, Opoji N 6°41’ 40 E 6°11’ 47.6”) into eastern Ondo State (Ifon N 6° 55’ 30.6” E 5° 46’ 34.5”, Okeluse N 6° 47’ 1.0” E 5° 35’ 10.9”, Owo N 7°12’ 28.2” E 5° 35’ 04.2”) (Figure 1). Using Sherman traps, all localities in Edo state were sampled for small mammals in July 2014. Ebudin and Ekpoma were additionally sampled 7 times during various seasons of the year up till April 2016. Within Ondo State, Ifon and Okeluse were trapped in October 2015 and Owo in May 2019.

Our trapping across the larger towns (Ekpoma ≈ 69.5 km^2^, Ifon 13.6 km^2^ and Owo 155.97 km^2^) aimed to cover as wide an area as possible, comprising distinct addresses with and without a history of confirmed Lassa fever cases. These sampling points, for which rodents were trapped indoors and in proximal environs outdoors, include what we categorized as private- or communal residences. Private residences generally consist of one-apartment buildings. A communal residence refers to a building that contains several apartments (such as a student hostel), living quarters amidst a public facility such as a market place, or a homestead regarded as part of a larger town (e.g. Eguare Egoro on the immediate outskirts of Ekpoma). Ekpoma (26 sampling points), Ifon (10 sampling points) and Owo (13 sampling points) are more metropolitan in comparison to the other four smaller localities trapped in this study, and form part of the major federal highway system of “Trunk A” roads [30].

For the smaller towns and villages (Ebudin ≈ 2.14 km^2^, Okeluse 3.47 km^2^, Okhuesan 3.9 km^2^ and Opoji 6.82 km^2^) trapping was carried out indoors across randomly selected houses (as opposed to particularly defined addresses) that formed a transect through the locality. Other transects were laid outdoors in domestic surroundings and wild vegetation. These smaller localities are accessible through secondary or tertiary roads (Figure 1). Traps were set for at least three nights per sampling visit to each locality in this study. A total effort of 14,988 trap-nights (i.e. number of traps set * number of nights) was expended, allocated according to locality as shown in Table 1.

**Table 1.** Distribution of *Mastomys* rodents PCR-positive for LASV across the Edo-Ondo area.

	**Edo State**				**Ondo state**			
Locality (No. of trap-nights)	**Ebudin** (8,190)	**Ekpoma** (4,101)	**Okhuesan** (486)	**Opoji** (480)	**Ifon** (450)	**Okeluse** (519)	**Owo** (792)	Total
Sampling date	July 2014 to April 2016	July 2014 to April 2016	July 2014	July 2014	Oct 2015	Oct 2015	May 2019	
*M. natalensis*, No. PCR positive/No. captured	18/131	14/57	1/27	–/3	4/23	37/49	13/28	90/318
*M. erythroleucus*, No. PCR positive/No. captured	10/68	1/5	–/–	–/–	–/3	–/–	–/–	12/76
* *								**102**/**394**

Only *Mastomys* rodents, initially identified by external morphology and body measurements [31] and later confirmed by DNA sequencing (see below), were PCR-positive for LASV. Thus, just specimens from this genus feature in our present report. The total composition of small mammal species captured and other ecological aspects of our investigations will be reported separately.

### 2.2. Molecular techniques

Upon euthanasia, 3 kinds of samples were obtained in the field from each individual rodent for LASV PCR testing. Cardiac puncture was carried out using a hypodermic syringe and whole-blood aliquots were made in two tubes: the first for primary LASV screening at the Irrua Specialist Teaching Hospital, Edo State, Nigeria (ISTH), and the second for confirmatory testing at the Bernhard Nocht Institute for Tropical Medicine, Hamburg, Germany (BNI). A third line of samples, obtained after dissection, consisted of visceral organs (i.e. liver, spleen and kidney, each in a separate tube).

A new syringe and fresh set of dissection tools were used, with working surfaces cleaned and disinfected, between the necropsy of each rodent. Blood and organs were stored at −20°C or −80°C and tubes were never re-opened until samples were drawn from them during the actual assays in the respective laboratories mentioned. RNA was extracted from whole-blood of *Mastomys* rodents (and visceral organs where appropriate) using the QIAamp Viral RNA kit (Qiagen GmbH, Germany). Organs, when used, were homogenized in Lysing Matrix D tubes (MP Biomedicals Inc., USA).

Rodents were screened by gel-based Reverse-Transcription PCRs targeting portions of the LASV genome belonging to the GPC (300 bp [32]) and L segments (300 bp [33]). Primers and amplification regimes are listed in Supplementary Material I. Comparison of PCR runs from independent blood samples in separate laboratories enabled us gauge the reliability of our LASV screening results. We included amplification of LASV from a visceral organ sample (usually liver) as a third option for verification for localities like Okeluse and Owo, where the *Mastomys* recorded an extraordinarily high prevalence and featured certain individuals with highly similar virus sequences (see Results). All in all, the veracity of each LASV sequence generated in this study was based on testing of multiple samples from each rodent in different laboratories. 873 nt of the GPC- and 768 nt of the NP-gene from PCR-positive extracts were sanger-sequenced and concatenated, resulting in a sequence comprising 1,641 nt which we refer to hereafter as the S fragment (Supplementary Material I).

As *Mastomys* specimens can be difficult to sort unambiguously into species using only external morphology, this task was facilitated by Cytochrome *b* DNA sequencing of visceral organs (primers and protocols follow Ducroz, Volobouev [34], Supplementary Material I).

Submissions were made to GenBank (www.ncbi.nlm.nih.gov) for LASV variants (accession numbers OQ556910 to OQ557079) and *Mastomys* Cytochrome *b* sequences (OQ656639 to OQ656649) originating from this study (Supplementary Material II).

### 2.3. Phylogenetic analysis

Our total data set of LASV genetic sequences consists of:

- S fragments from 111 variants sequenced from *Mastomys* rodents, of which 85 are new sequences sampled during 2014–2019 across seven localities (Figure 1, Supplementary Material II). Twenty-six of these 111 variants from Ekpoma have already been published in preliminary phylogenetic investigations [25,26].
- S fragments from 82 variants sequenced in *Homo sapiens* living in Edo and Ondo states. We selected sequences from the same localities as our rodent-obtained LASV variants. These human-sourced sequences were sampled recently by Ehichioya et al. [20] and Klitting et al. [35]. Some outgroup sequences from Benin city, Irrua, and Uromi were also added (see the list in Supplementary Material II).

The phylogenies were inferred by the Bayesian Markov Chain Monte Carlo (MCMC) method, implemented in BEAST software (https://beast.community) and developed in 2 models.

Model 1: To evaluate LASV clade distribution, we included our 111 rodent-derived sequences from the Edo and Ondo area (representing sub-lineage 2 g) plus 2 human-derived sequences from Anambra and Koji as an outgroup (representing sub-lineage 2f; Ehichioya et al. [20]). This analysis was thus conducted running 113 variants.

In BEAUTI, the parameters were:

- One alignment including the partial GP (873 nt) and NP (768 nt), concatenated in a chimeric open reading frame S fragment;
- Tip dates at the nearest day;
- Substitution model as GTR + gamma and codon partition with positions 1, 2, 3;
- Strict clock;
- Coalescent tree with a constant size population;
- MCMC = 150 M, echo states, and log parameters every 100,000.

Model 2: To estimate times of divergence in both *Mastomys* and humans, we added 82 human-derived sequences to our rodent-derived ones. This was conducted running 193 variants.

In BEAUTI, the parameters were:

- One alignment including the partial GP (873 nt) and NP (768 nt), concatenated in a chimeric open reading frame S fragment;
- Twelve taxa were defined: *Mastomys natalensis* Ebudin, *Mastomys natalensis* Eguare-Egoro, *Mastomys natalensis* Ekpoma, *Mastomys natalensis* Ifon, *Mastomys natalensis* Okeluse, *Mastomys natalensis* Owo, *Mastomys erythroleucus* Ebudin, *Homo* Ekpoma, *Homo* Ifon, *Homo* Okeluse, *Homo* Owo, *Homo* Uromi. Details are contained in the Supplementary Material II;
- Tip dates at the nearest day;
- Substitution model as GTR + gamma and codon partition with positions 1,2,3;
- Strict clock;
- Coalescent tree with a constant size population;
- MCMC = 100 M, echo states, and log parameters every 100,000.

The xml files issued from BEAUTI were run in BEAST, the log files checked in TRACER, and consensus trees were visualized through FigTree (BEAST packages, https://beast.community/programs).

## 3. Results

### 3.1. LASV prevalence and resolution of phylogenetic clades

A total of 417 *Mastomys* were captured: 347 *M. natalensis* and 70 *M. erythroleucus*. Table 1 shows the geographic distribution of these species. *Mastomys natalensis* was present in all localities while *M. erythroleucus* occurred mainly in Ebudin, with few individuals also detected in Ekpoma and Ifon. *Mastomys natalensis* was PCR-positive for LASV in Ebudin (14%, 18/131), Ekpoma (25%, 14/57), Okhuesan (4%, 1/27), Ifon (17%, 4/23), Okeluse (prevalence 76%, 37/49), and Owo (46%, 13/28); whereas *M. erythroleucus* was also positive in Ebudin (16%, 10/68) and Ekpoma (20%, 1/5).

**Figure 2.**
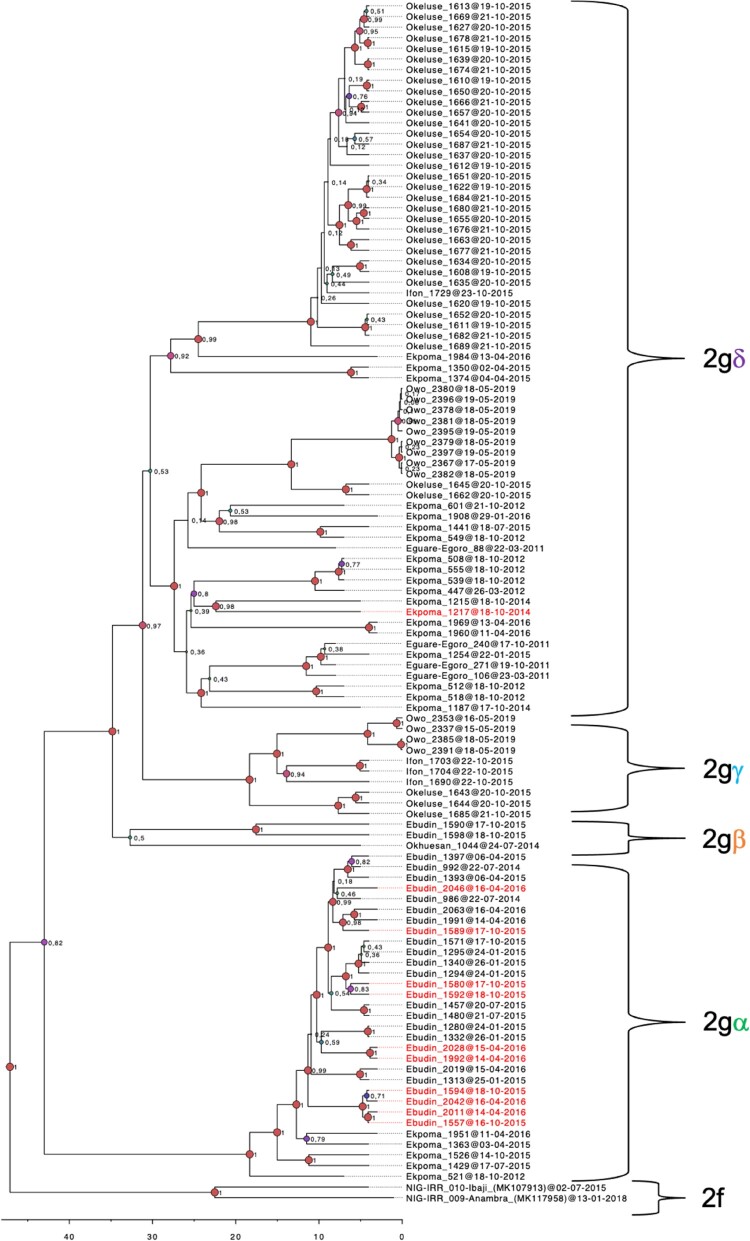
Phylogenetic tree of LASV sequences obtained from *Mastomys* rodents in the Edo-Ondo area. The analysis is based on a concatenated “S” sequence which includes partial glycoprotein and nucleoprotein fragments from 111 rodents. Two human-derived sequences were used as outgroups to show the sub-lineage 2f. Labels 2gα-δ identify ingroup clades. Ingroup sequences derived from *M. natalensis* are in labelled black, while those from *M. erythroleucus* are in red.

The LASV sequences from our *Mastomys* rodents in the Edo-Ondo area were resolved into four clades with > 0.8 nodal support within sub-lineage 2 g of lineage II (Figure 2). Clade 2g-alpha (2gα) comprises the majority of sequences from Ebudin (12) and five from Ekpoma. Clade 2g-beta (2gβ) consists of two Ebudin sequences and the sole one from Okhuesan. Within 2gβ, the Okhuesan sequence has a deeper node, but this separation showed only 0.5 nodal support. Thus, LASV variants from clades 2gα and 2gβ, which are basal to the remaining clades, circulate within Edo state (i.e. in sites to the south-east of our study area) (Figures 1 and 2). Variants from clade 2g-gamma (2gγ) circulate across Ifon, Okeluse and Owo, but are limited only to Ondo State. Variants from clade 2g-delta (2gδ) are the most widespread geographically, distributed across all localities sampled in Ondo State but also extending into Ekpoma within Edo State. This clade appears divided into two clusters, but again, the nodal support for this bifurcation is low (0.53).

Smaller localities (< 10 km^2^; Ebudin, Okhuesan, Okeluse) had a higher mean percentage sequence similarity (96.37–97.54%), while the bigger towns (Ekpoma, Ifon, Owo) possessed relatively lower sequence similarity (93.87–96.65%) (Table 2). Ekpoma showed the lowest average sequence similarity (93.87%).

**Table 2.** Mean percentage similarity (± standard deviation) between LASV sequences within each locality.

Owo *n* = 13 (155.97 km^2^)	Ekpoma *n* = 29^a^ (69.5 km^2^)	Ifon *n* = 4 (13.6 km^2^)	Okeluse *n* = 37 (3.47 km^2^)	Ebudin *n* = 28 (2.14 km^2^)
96.65% (± 3.18)	93.87% (± 1.79)	95.3% (± 2.68)	97.54% (± 2.15)	96.37% (± 3.6)

The sites are arranged according to size. Okhuesan is left out because it had only one LASV-positive rodent. ^a^This includes sequences recovered in this locality during the present study (2014–2016) and previously (2011–2012), published by Olayemi et al. [25].

### 3.2. Fine-scale circulation within towns

**Figure 3.**
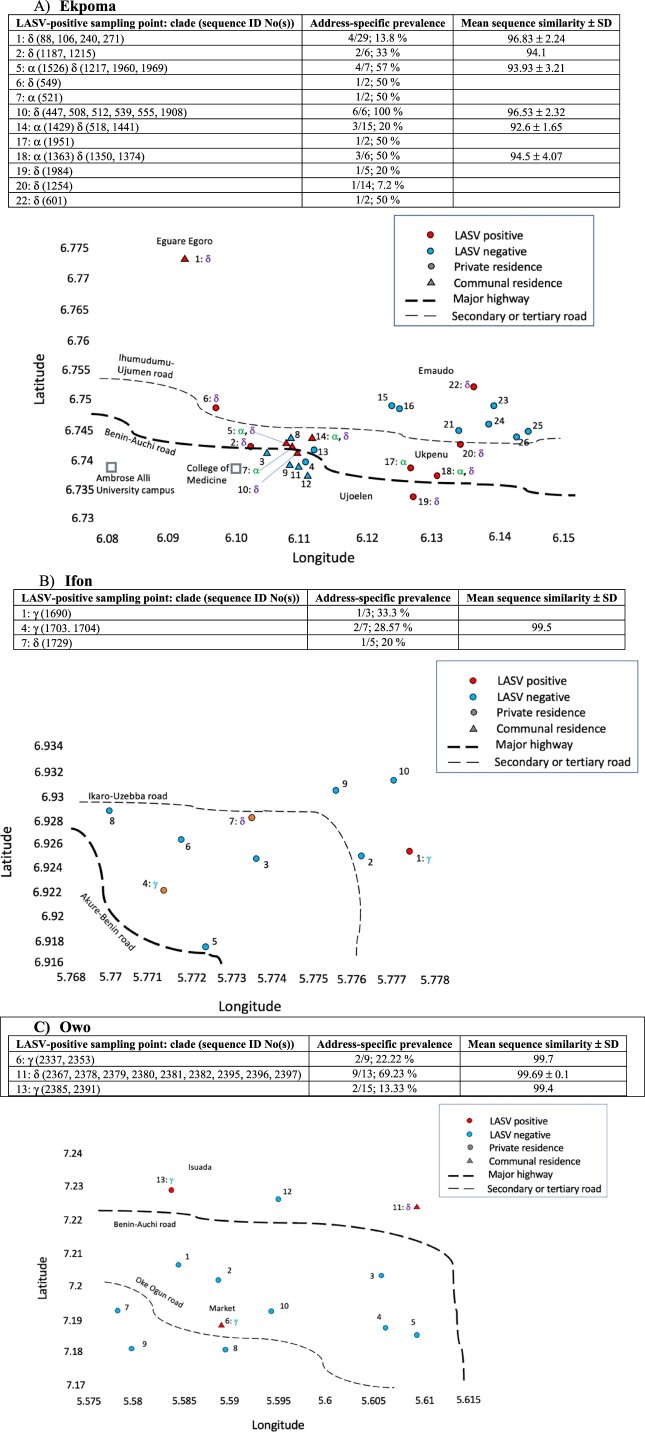
Fine-scale LASV clade distribution within the larger-sized localities. Serial numbers represent sampling points (1–26 for Ekpoma, 1–10 for Ifon and 1–13 for Owo). Red sampling points within each map indicate where LASV-positive *Mastomys* were captured, with corresponding virus clades beside the sampling point number (e.g. 1: α, β, γ). Above each locality map appear details concerning the distribution of LASV sequences among sampling points and clades (e.g. 1: δ (88, 97, 109, 240, 271)), the virus prevalence in *Mastomys* at each LASV-positive address, and the mean sequence similarity. Circular sampling points denote private residences, and triangles communal residences. Data from 2011 to 2012 [25,29] are included for Ekpoma.

Figure 3 depicts fine-scale spatial distribution of LASV clades within the larger-sized localities: Ekpoma, Ifon and Owo. Twenty-six addresses were sampled in Ekpoma (Figure 3). These form three spatially recognizable zones: Eguare Egoro (sampling point 1), a homestead on the western fringe of town; private residences and student hostels in the vicinity of the main campus of Ambrose Alli University and its College of Medicine (sampling points 2–14); and private residences around Ujoelen-Ukpenu-Emaudo (sampling points 15–26) in the eastern portion of Ekpoma.

About half (12/26) of all these addresses had LASV-positive *Mastomys*. Seven out of the 12 LASV-positive sampling points were private residences. Only a single LASV clade (either 2gα or 2gδ) was detected in each of these 7 private residences, except at sampling point 18. Also, only one *Mastomys* rodent was LASV-positive in each of these private residences, except at sampling points 2 and 18. On the other hand, addresses with the highest LASV prevalence in Ekpoma were student hostels (i.e. sampling point 10 (100% prevalence, 6/6) and sampling point 5 (prevalence 57%, 4/7)). Student hostels also displayed the greatest LASV heterogeneity, with the lowest mean similarities between variants recorded at sampling points 14 (92.6 ± 1.65) and sampling point 5 (93.93 ± 3.21).

In Ifon, three out of ten sampling points had LASV-positive *Mastomys*; all three being private residences. LASV clade 2gδ was detected at one of these addresses, while clade 2gγ circulated in the other two. Three also, out of 13 sampling points, were LASV-positive in Owo. Only one out of the three (serial number 13, in Isuada) was a private residence, bearing LASV clade 2gγ. Sampling point 6, also harbouring clade 2gγ, is an address within a major market in the heart of Owo. Sampling point 11, a student hostel, contained clade 2gδ. Notably, *Mastomys* in this student hostel comprised the bulk of LASV-positive individuals detected in Owo (9 out of 13). Additionally, LASV variants with almost exact (99.8%) sequences were observed between pairs of *Mastomys* from this hostel (identification numbers 2380 and 2396, 2378 and 2381, 2367 and 2382, 2379 and 2397 for example; Figure 2, Supplementary material III). This suggests a very recent or ongoing virus jump among the individuals concerned at the time of capture. Only a single LASV clade was present in each virus-positive address detected in Ifon and Owo.

### 3.3. LASV clustering according to host species and temporal emergence

Apart from *Mastomys natalensis*, 11 *M. erythroleucus* were also LASV-positive in this study; one in Ekpoma and the other ten in Ebudin (Table 1). The infected *M. erythroleucus* in Ekpoma (identity number 1217) was detected in a student hostel (sampling point 5; Figure 3). All other LASV-positive individuals captured at this address were *M. natalensis*. Additionally, the LASV variant found in the Ekpoma *M. erythroleucus* (which belongs to clade 2gδ) has as its closest relative a *M. natalensis*-derived LASV sequence (identification number 1215) from sampling point 2, a private residence in the area (Figures 2 and 3). All LASV sequences from *M. erythroleucus* in Ebudin fall within clade 2gα, some of them forming noticeable sub-clades. Nevertheless, whether appearing as a single sequence or clusters within clade 2gα, *M. erythroleucus*-derived variants always had a LASV sequence from *M. natalensis* as their closest relative. Thus, LASV variants detected in *M. natalensis* and *M. erythroleucus* are intertwined within clades 2gα and 2gδ in our phylogenetic tree, and do not appear to cluster to any significant extent according to the specific rodent from which they were obtained.

**Figure 4.**
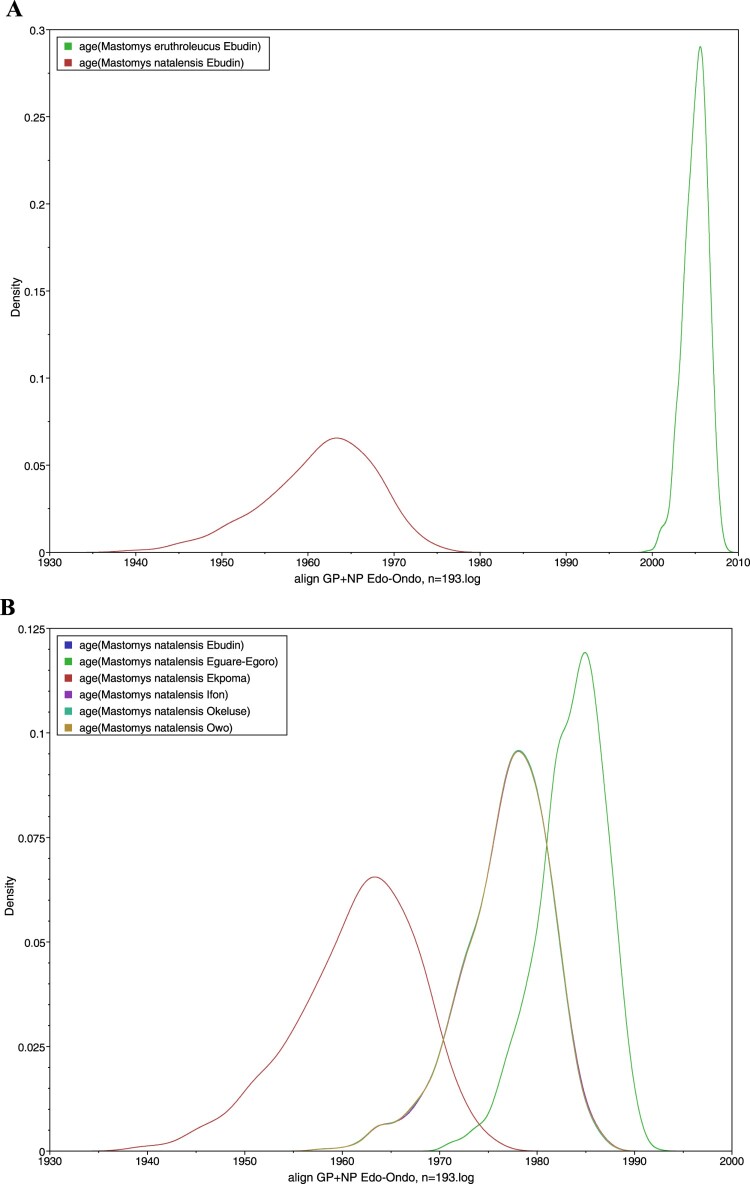
Estimated dates of emergence for LASV sequences obtained from *Mastomys* rodents. (A) *M. natalensis* and *M. erythroleucus* in Ebudin. (B) *M. natalensis* in various localities.

However, our time-calibrated analyses demonstrated that the LASV sequences derived from *M. erythroleucus* in Ebudin emerged significantly more recently (approximately during 2005) compared to those from this same locality obtained from *M. natalensis* (estimated to emerge around 1961) (Figure 4(A)). The time-calibrated analyses also showed that LASV sequences derived from *M. natalensis* in Ebudin and Ekpoma (excluding Eguare-Egoro) had earlier emergence dates (averaged at 1961) than those from Ifon, Okeluse and Owo (1977) and Eguare-Egoro (1983), suggesting a broadly east-to-west progression of the virus among rodents (Figure 4(B), Table 3). The human-derived LASV sequences from Edo- and Ondo States which we included to expand our phylogeny (Supplementary material IV) largely clustered with those acquired from rodents that belong to the same localities. The clade topology was broadly similar to the phylogenetic tree in Figure 2 which featured only virus sequences sourced from *Mastomys*.

**Table 3.** Mean ages and 95% HPD intervals of LASV in *Mastomys natalensis* and in *Homo sapiens* per locality.

Locality	Age, *M. natalensis*	Age, *H. sapiens*
Ebudin	1961 (1948–1973)	Not available
Eguare-Egoro	1983 (1976–1989)	Not available
Ekpoma	1961 (1948–1973)	1961 (1948–1973)
Ifon	1977 (1967–1985)	1977 (1967–1985)
Okeluse	1977 (1967–1985)	1979 (1971–1986)
Owo	1977 (1967–1985)	1961 (1948–1973)
Uromi	Not available	1974 (1965–1983)

**Figure 5.**
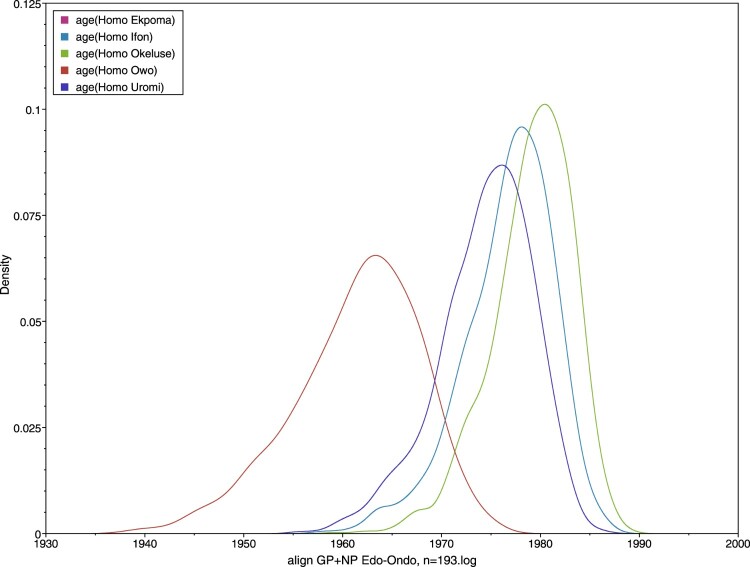
Estimated dates of emergence for LASV sequences obtained from humans.

Another key feature common to both trees was the unstable position of sequence 1044 from Okhuesan. In Figure 2 it clustered to clade 2gβ sequences from Ebudin with only 0.5 nodal support. In the phylogeny containing human-derived sequences, it fell between the 2gβ sequences and a cluster formed by those from Uromi; again, with weak support (0.59). Temporally, the human-derived LASV sequences have largely similar emergence times with those from *M. natalensis* in Ekpoma (dated ≈ 1961), Ifon (1977) and Okeluse (1979) (Table 3, Figure 5). This again suggests an essentially east-to-west migration of the virus; or at least a radial spread originating from Ekpoma, if the human-derived sequences from Uromi (1974) (a locality 20 km east of Ekpoma; see Figure 1) are considered. An exception to the general trend is Owo, where the human-derived sequences were estimated to be strikingly older than the rodent-derived sequences (1961 vs. 1977 in Table 3).

## 4. Discussion

Investigations on the molecular epidemiology of LASV within Nigeria have largely involved virus sequences obtained from humans [16,20] and knowledge regarding LASV variability and transmission patterns within natural rodent reservoirs is only incipient [26]. Here, our results from the most endemic area for Lassa fever within Nigeria increase insight into the emergence and circulation of rodent-borne LASV variants between and within localities, and also amongst host species (*Mastomys natalensis* and *M. erythroleucus*). The primary LASV rodent reservoir, *M. natalensis*, was present in all the localities we trapped, commensurate to the fact that this species is known to occur all over sub-Saharan Africa [36]. Virus prevalence was discovered to reach extraordinarily elevated proportions within *M. natalensis* in a site like Okeluse (76%). More extensive clinical and seroprevalence surveys can help gauge the impact of this among humans in this particular locality. All LASV sequences obtained from our *Mastomys* rodents in Edo and Ondo states fell within lineage II, corresponding also to sub-lineage 2 g as recently described by Ehichioya et al. [20].

Investigations on spatial LASV evolution in Guinea and Sierra Leone have reported a combination of stationary circulation within, and virus movement between, villages [23,37]. Similarly, in this study, certain variants are relatively limited in circulation; such as those in clade 2gγ, confined to Ondo state, or clades 2gα and 2gβ, which are restricted to Edo state. Clade 2gδ, on the other hand, is distributed into both Edo and Ondo states. In countries like Guinea, Lassa fever has long been regarded as a disease of the rural areas [24,38]. Our findings show that, at least within Nigeria, larger, relatively urban localities play an important part in the propagation of the virus. That more metropolitan towns contribute to human-aided dissemination of LASV-carrying *Mastomys* is illustrated in Ifon, where only 4 LASV-positive *M. natalensis* were detected, but the virus sequence similarity is actually second lowest among all localities.

Still, Ekpoma displays the greatest diversity and is the most cosmopolitan and polyphyletic in terms of LASV variant origin. Congruent with these properties is its comparatively large size, its connection to a major highway and its position in the middle of the Edo-Ondo Lassa fever belt. This contributes to the notion that human-assisted transportation of rodents or contaminated surfaces play a significant role in LASV dissemination, with the potential that the virus can be introduced via this route into areas that are currently non endemic. Interestingly, our survey also demonstrates that, within municipalities like Ekpoma and Owo, communal, transitory accommodation such as student hostels (featuring a high turnover of inhabitants who come and go from far and wide) serve, in effect, as hubs of anthropologically-aided, rodent-borne LASV variant admixture and diffusion. We believe the greater genetic diversity documented in Ekpoma was not necessarily due to surplus sampling effort, as the two clades ultimately recovered in Ekpoma (2gα and 2gδ) were already detected in the first sampling sessions during 2011–2012 (Figure 2). Moreover, the sequence similarity recorded in Ebudin also remained high in spite of longitudinal sampling.

Even more remarkable is our confirmation by PCR screening that *M. erythroleucus* is also a LASV reservoir within the Edo-Ondo axis, as suggested recently by serological data [28]. More so, this is the first time lineage II is being detected in *M. erythroleucus*. Olayemi et al. [27] beforehand discovered other LASV lineages in this same rodent: lineage III toward central Nigeria and lineage IV around coastal Guinea. LASV PCR-positive *M. natalensis* and *M. erythroleucus* were detected within the same locality (as occurred in Ebudin and, to a lesser extent, Ekpoma). The LASV variants sequenced from both multimammate mice did not cluster according to host species. This suggests that, currently, within Ebudin and Ekpoma, LASV, unconstrained genetically, jumps horizontally between individuals of separate *Mastomys* taxa. *Mastomys natalensis* and *M. erythroleucus* are clearly separate species, possessing chromosome numbers 2*n* = 32 and 2*n* = 38 respectively [36]. Nevertheless, they are sibling taxa and likely present analogous physiological environments for LASV maintenance [14]. Evidence is being provided that increasingly repudiates the one-genotype-one-rodent concept concerning the relationship between viruses and their rodent reservoir species [39,40].

However, the significantly recent emergence date of LASV sequences within *M. erythroleucus* in Ebudin is consistent with the mounting encroachment of this principally savanna-dwelling multimammate mouse into the degraded forest within southern Nigeria [41]; where LASV-bearing *M. natalensis* populations are already present in sites around the Edo-Ondo area. Such a dynamic that involves the proliferation of LASV geographically but also between *Mastomys* species represents vital information for the epidemiology and control of Lassa fever; as sympatric populations of *M. natalensis* and *M. erythroleucus*, many at the risk of becoming infected, are spread across Nigeria and Western Africa [14].

Furthermore, with regard to temporal emergence, the fact that LASV variants in *M. natalensis* from Edo are essentially ancestral to those from Ondo State is in line with the concept of east-to-west advancement of the virus through southern Nigeria at the regional level [20]. Human-derived LASV variants entered into the analyses conformed generally to this emergence pattern but also revealed certain departures from the overall trend at the local level. Specifically, these involve the younger emergence date of Uromi (≈ 1974) in Edo State compared to that of Ekpoma (1961), suggesting a minor progression eastward; and Owo, where the virus appears to have arrived much earlier in humans (1961) than rodents (1977). The incongruity in Owo hints at independent, host-specific introductions of the virus into this locality and/or a reverse zoonosis, where infections pass (through the contaminated environment) from humans to rodents. This phenomenon was recently pointed out preliminarily [26], where *Mastomys*-borne LASV sequences in Ekpoma were compared to human-sourced variants from Edo (identifiable at the time only by state, not locality) [19]. Since then, an increase in the number of genetic sequences with reliable date-and-locality stamps obtained from rodents (this study) and humans [20,35] provide a more refined picture upon which the evolutionary history and molecular epidemiology of LASV in the Edo-Ondo area (especially where it concerns the rodent—human interface) can be continually developed.

## 5. Conclusion

Our study provides important information regarding point-prevalence, patterns of spatial variability and temporal emergence of rodent-borne LASV in the Edo-Ondo hotspot. It also provides evidence of active exchange of the virus between sympatric *Mastomys* host species. This augments insight relating to the zoonotic risk of Lassa fever in this endemic area, but additionally points out potential avenues for future emergence. Extension of our longitudinal sampling, accompanied by full genome sequencing of recovered LASV variants, will facilitate subsequent research into how the variability we’ve characterized in this study could evolve through time. Building on our data will also furnish knowledge at a finer scale concerning how these variants are transmitted between rodents and humans. The genetic range of LASV variants realized from our ecological surveys should assist current efforts to develop a Lassa fever vaccine [10]; still, rodent control remains the strongest option for the prevention of this disease.

## Supplementary Material

Supplemental Material
