# Supplementary material for: Circulation of Lassa virus across the endemic Edo-Ondo axis, Nigeria, with cross-species transmission between multimammate mice
Source: Emerg Microbes Infect. 2023 Jun 8;12(1):2219350. doi: 10.1080/22221751.2023.2219350 (PMC10251791; doi:10.1080/22221751.2023.2219350)
Supplement: Supplemental Material [file TEMI_A_2219350_SM7673.zip › Supplementary material I.docx]

| **LASV genetic fragment** | **Primer sequence** | **PCR temperature profile** | **Reference** |
| --- | --- | --- | --- |
| GPC, ≈ 300 bp | Forward:  36E2: ACC GGG GAT CCT AGG CAT TT  Reverse:  LVS-339: GTT CTT TGT GCA GGA MAG GGG CAT KGT CAT | 50 °C for 30 min  95 °C for 15 min  95 °C for 30 sec  45x  52 °C for 30 sec  72 °C for 30 sec | [Olschlager, Lelke [32]](#_ENREF_30) |
| GPC, ≈ 900 bp | Forward:  OWS0001: 5´-GCGCACCGGGGATCCTAGGC-3  Reverse:  OWS 1000: 5´-AGCATGTCACAGAAYTCYTCATCATG-3´ | 50 °C for 30 min  95 °C for 15 min  95 °C for 20 sec  45x  52 °C for 30 sec  72 °C for 1 min | [Ehichioya, Hass [13]](#_ENREF_41) |
| L, ≈ 300 bp | Forward:  LVL3359A-plus: AGAATTAGTGAAAGGGAGAGCAATTC  LVL3359D-plus: AGAATCAGTGAAAGGGAAAGCAATTC LVL3359G-plus: AGAATTAGTGAAAGGGAGAGTAACTC Reverse:  LVL3754A-minus: CACATCATTGGTCCCCATTTACTATGATC  LVL3754D-minus: CACATCATTGGTCCCCATTTACTGTGATC | 50 °C for 30 min  95 °C for 15 min  95 °C for 20 sec  45x  52 °C for 30 sec  72 °C for 1 min | [Vieth, Drosten [33]](#_ENREF_31) |
| NP, ≈ 780 bp | Forward:  LVSnig 1669+: 5'-TATATTGAGTCCTCCTGACACAG-3'  Reverse:  LVSnig 2511-: 5'-TGTTGGAGACCATCAAGGTT-3' | 50 °C for 30 min  95 °C for 15 min  95 °C for 20 sec  35x  52 °C for 30 sec  72 °C for 1 min | [Olayemi, Adesina [26]](#_ENREF_24) |
| ***Mastomys* genetic fragment** |  |  |  |
| Cytochrome b, ≈ 1,100 bp | Forward:  L7: 5'ACC AAT GAC ATG AAA AAT CAT CGT T-3'  Reverse:  H15915: 5'-TCT CCA TTT CTG GTT TAC AAG AC-3' | 95 °C for 3 min  95 °C for 20 sec  35x  55 °C for 20 sec  72 °C for 60 sec | [Ducroz, Volobouev [34]](#_ENREF_32) |
