# Supplementary material for: Circulation of Lassa virus across the endemic Edo-Ondo axis, Nigeria, with cross-species transmission between multimammate mice
Source: Emerg Microbes Infect. 2023 Jun 8;12(1):2219350. doi: 10.1080/22221751.2023.2219350 (PMC10251791; doi:10.1080/22221751.2023.2219350)
Supplement: Supplemental Material [file TEMI_A_2219350_SM7673.zip › Supplementary material IV.docx]

**Supplementary Material IV: Phylogenetic tree of LASV sequences obtained from *Mastomys* rodents and humans in the Edo-Ondo area.** The analysis is based on the S sequence (comprising partial glycoprotein and nucleoprotein fragments) from 111 rodents and 82 humans living in Edo and Ondo states. The tree is rooted with 2 human-derived sequences from Anambra and Koji. Sequences derived from *M. natalensis* are labelled in black, while those from *M. erythroleucus* are in red. Human-derived sequences are also labelled in black, but bear labels with the prefix “NIG-IRR…”
